# Exosomal Communication Between Cumulus–Oocyte Complexes and Granulosa Cells: A New Molecular Axis for Oocyte Competence in Human-Assisted Reproduction

**DOI:** 10.3390/ijms26115363

**Published:** 2025-06-03

**Authors:** Charalampos Voros, Diamantis Athanasiou, Despoina Mavrogianni, Antonia Varthaliti, Kyriakos Bananis, Antonia Athanasiou, Aikaterini Athanasiou, Georgios Papadimas, Athanasios Gkirgkinoudis, Ioannis Papapanagiotou, Kyriaki Migklis, Dimitrios Vaitsis, Aristotelis-Marios Koulakmanidis, Dimitris Mazis Kourakos, Sofia Ivanidou, Maria Anastasia Daskalaki, Marianna Theodora, Panagiotis Antsaklis, Dimitrios Loutradis, Georgios Daskalakis

**Affiliations:** 1 1st Department of Obstetrics and Gynecology, ‘Alexandra’ General Hospital, National and Kapodistrian University of Athens, 80 VasilissisSofias Avenue, 11528 Athens, Greece; depy.mavrogianni@yahoo.com (D.M.); antonia.varthaliti@hotmail.com (A.V.); tgkirgki@gmail.com (A.G.); aristoteliskoulak@gmail.com (A.-M.K.); md181341@students.euc.ac.cy (M.A.D.); martheodr@gmail.com (M.T.); panosant@gmail.com (P.A.); gdaskalakis@yahoo.com (G.D.); 2 IVF Athens Reproduction Center V. Athanasiou, 15123 Maroussi, Greece; diamathan16@gmail.com (D.A.); antoathan16@gmail.com (A.A.); diamathan17@gmail.com (A.A.); 3 King’s College Hospitals NHS Foundation Trust, London SE5 9RS, UK; kyriakos.bananis@nhs.net; 4 Athens Medical School, National and Kapodistrian University of Athens, 15772 Athens, Greece; dr.georgepapadimas@gmail.com (G.P.); gpapamd@hotmail.com (I.P.); kyriaki.migklis@gmail.com (K.M.); vaitsisdim@gmail.com (D.V.); mazisdimitris@gmail.com (D.M.K.); info@ivanidou.gr (S.I.); loutradi@otenet.gr (D.L.); 5 Fertility Institute-Assisted Reproduction Unit, Paster 15, 11528 Athens, Greece

**Keywords:** exosomal microRNAs, follicular fluid, cumulus cells, oocyte competency, extracellular vesicles, IVF, ARTs, miRNA signaling

## Abstract

Exosomal microRNAs (ex-miRs), encapsulated in extracellular vesicles (EVs), play a vital role in facilitating paracrine communication among granulosa cells (GCs), cumulus cells (CCs), and the oocyte inside follicular fluid (FF). These small non-coding RNAs are crucial for regulating folliculogenesis, oocyte maturation, and early embryonic development via modulating intracellular signaling networks. Dysregulation o has been associated with reproductive disorders such as polycystic ovarian syndrome (PCOS), diminished ovarian reserve (DOR), and inadequate ovarian response (POR), impacting oocyte quality and fertility outcomes. This narrative review consolidates molecular data from current human and animal studies regarding ex-miR expression patterns, functional targets, and pathway involvement within the context of assisted reproductive technologies (ARTs). A literature-based analysis was undertaken, focusing on signaling pathways, pathogenic processes, and clinical implications. Specifically, ex-miRs—such as miR-21, miR-34c, miR-143-3p, miR-155-5p, miR-339-5p, and miR-424-5p—were identified as regulators of critical pathways including phosphoinositide 3-kinase (PI3K)–AKT, ERK1/2, TGF-β/SMAD, and Rb–E2F1. These ex-miRs regulate apoptosis, glycolysis, mitochondrial function, and cell cycle expansion to influence oocyte competence. Pathological patterns in PCOS and POR are associated with altered ex-miR expression that disrupts metabolic and developmental signaling. Research utilizing animal models confirmed that modifications in EV-associated miRNA influence in vitro maturation (IVM) efficiency and blastocyst quality. Ex-miRs serve as intriguing non-invasive biomarkers and potential therapeutic targets for ARTs. Their mechanical involvement in oocyte and follicular physiology positions them for integration into forthcoming precision-based infertility therapies. For its implementation in reproductive medicine, EV profiling requires standardization and further functional validation in clinical environments.

## 1. Introduction

The efficacy of assisted reproductive technologies, including in vitro fertilization (IVF), is predominantly contingent upon the quality of the oocyte. Oocyte competence, defined as the ability of the oocyte to resume meiosis, undergo fertilization, and facilitate embryo development, is governed by a meticulously regulated interaction between the oocyte and the adjacent somatic cells of the ovarian follicle, especially cumulus and granulosa cells [1,2]. These cells provide a dynamic and metabolically active milieu that regulates folliculogenesis, steroidogenesis, and oocyte maturation via bidirectional communication [3].

The cellular communication within the follicular niche has conventionally been interpreted through gap junctions, endocrine signals, and paracrine signaling. Recent data has revealed that exosomes, a subtype of extracellular vesicles (EVs), serve as crucial regulators of intercellular signaling in the ovary. Exosomes are lipid-bilayer vesicles derived from endosomes, generally measuring 30–150 nm in diameter, and contain a varied array of molecular constituents, including microRNAs, mRNAs, long non-coding RNAs (lncRNAs), proteins, and lipids [4,5]. Their biological purpose consists of transferring this cargo from donor to recipient cells, hence altering the phenotypic and gene expression of the target cells in a precise and context-dependent manner [6].

Exosomes are notably present in human follicular fluid, with their concentration and molecular composition differing based on follicular size, hormonal environment, and pathological states [7]. Numerous studies have demonstrated that the exosomal miRNA profile in follicular fluid correlates with oocyte quality, fertilization success, and embryo development. For example, mir-21, recognized for its anti-apoptotic and pro-survival roles, has been positively correlated with mature oocytes and high-quality embryos [8]. Other miRNAs, including miR-92a, miR-130b, and miR-320a, exhibit substantial differences between successfully fertilized oocytes and those that did not fertilize, indicating a possible function in oocyte competence and early embryogenesis [9].

Despite heightened interest in exosomes as diagnostic and therapeutic instruments in reproductive medicine, the literature remains disjointed, featuring diverse methodologies and aims. There is a lack of consensus on established methodologies for exosome isolation, characterization, and molecular profiling in reproductive organs [10]. Moreover, whereas several animal studies have elucidated the mechanistic effects of exosomal cargo on folliculogenesis and oocyte maturation, data related to humans are few and predominantly observational [11].

This narrative review aims to synthesize current human data about exosomal communication between cumulus–oocyte complexes (COC) and granulosa cells, focusing on its impact on oocyte quality and developmental competence. We will examine the biological origin and function of follicular exosomes, summarize findings from both normal and pathological conditions (including PCOS, DOR, and advanced maternal age), and discuss molecular mechanisms, particularly exosomal microRNAs, that may act as predictive biomarkers or therapeutic targets in ARTs. This study seeks to provide a novel perspective on oocyte competence by elucidating the role of exosomes in the follicular milieu, thereby advancing the long-term goal of integrating non-invasive, molecular diagnostics and therapies into clinical IVF practices.

## 2. Exosomes in the Follicular Environment: Biology and Mechanisms

### 2.1. Biogenesis, Cargo, and Uptake of Exosomes

Exosomes, originating from the endosomal pathway, transmit regulatory molecular cargo from donor to recipient cells, thereby facilitating intercellular communication inside the ovarian follicle. Key functions in follicular signaling include their nanoscale dimensions and ability to encapsulate bioactive molecules [12]. Their biogenesis commences with the inward invagination of the endosomal membrane during the maturation of early endosomes into late endosomes or multivesicular bodies. These entities amass intraluminal vesicles that are intended to transform into exosomes upon fusion with the plasma membrane and subsequent release into the extracellular environment [13]. The Endosomal Sorting Complex Required for Transport machinery, consisting of ESCRT-0, ESCRT-I, ESCRT-II, and ESCRT-III subcomplexes, along with accessory proteins like TSG101, VPS4, and Alix, regulates this process by facilitating membrane deformation, cargo selection, and vesicle scission within the maturing endosome [14]. Alternative ESCRT-independent mechanisms have been identified, including ceramide-mediated budding and lipid raft domains, along with tetraspanins such as CD9, CD63, and CD81, which promote membrane curvature and vesicle stabilization, and are frequently utilized as exosomal surface markers in analytical platforms [15].

Exosomes engage with adjacent cells via multiple mechanisms, such as clathrin-mediated or caveolin-mediated endocytosis, macropinocytosis, phagocytosis, and direct fusion with the plasma membrane, contingent upon the molecular composition of the exosomal membrane and the receptor profile of the recipient cell [12]. The uptake of exosomes is often facilitated by specific ligand-receptor interactions involving integrins, heparan sulfate, proteoglycans, and other adhesion molecules, which enhance the selective targeting and internalization of exosomes, thereby ensuring the functional transfer of their molecular cargo into the cytoplasm of the recipient cell.

The molecular cargo of exosomes is selectively sorted during their formation and comprises a diverse array of regulatory molecules that mirror the physiological or pathological condition of the donor cell, capable of modifying the transcriptomic, proteomic, and metabolic profiles of the recipient cell [16]. MicroRNAs, a well-studied component of exosomes, are small non-coding RNAs that regulate gene expression post-transcriptionally by binding to complementary sequences in the 3′ untranslated region of target mRNAs, leading to translational inhibition or transcript degradation [17].

Exosomes, alongside miRNAs, encompass mRNAs capable of being translated into functional proteins in recipient cells, as well as long non-coding RNAs like MALAT1 or H19, which may influence gene expression through chromatin remodeling, transcriptional interference, or miRNA sponging; however, their role in follicular physiology is not yet fully elucidated [18]. The protein composition of exosomes is crucial, encompassing molecular chaperones such as HSP70 and HSP90, which are involved in protein folding and stress responses; signal transduction mediators like annexins and 14-3-3 proteins that play roles in intracellular trafficking and apoptosis regulation; and metabolic enzymes and structural proteins that indicate the functional status of the follicular environment [19].

Exosomes transport bioactive lipids such as sphingomyelin, phosphatidylserine, and ceramide, which enhance membrane stability, facilitate vesicle formation, and influence intracellular signaling processes that impact oocyte competence and follicular cell viability [20]. The molecular profile of exosomes in the ovarian follicle reflects the condition of the cumulus–oocyte complex and adjacent granulosa cells, while also functioning as a dynamic medium for information exchange and coordination of processes essential for oocyte maturation, cytoplasmic competence, and subsequent embryonic development [21].

### 2.2. Molecular Pathways Regulated by Exosomal microRNAs in the Follicular Niche

Exosomal microRNAs (miRNAs) found in the ovarian follicular milieu are now recognized as important regulators of gene expression and intracellular signaling pathways that control follicular development, somatic cell function, and oocyte competence [22]. These ex-miRs, secreted by granulosa cells, cumulus cells, and possibly the oocyte itself, are actively transferred to neighboring cells via EV-mediated signaling. They are transmitted to nearby cells via active vesicle uptake mechanisms such as receptor-mediated endocytosis and membrane fusion [23].

Exosomal miRNAs engage with Argonaute proteins to form RNA-induced silencing complexes (RISCs), which then associate with complementary sequences in target mRNAs, leading to translational repression or mRNA degradation [24]. This technique enables accurate modulation of essential genes implicated in oocyte maturation, cumulus expansion, and follicular integrity. Research has underscored the functional significance of exosomal miRNAs in human reproduction, demonstrating correlations between their expression in follicular fluid and several IVF outcomes, including oocyte maturity, fertilization rates, embryo quality, and clinical pregnancy rates [21].

Activated AKT phosphorylates numerous downstream substrates, including the pro-apoptotic transcription factors FOXO1 and FOXO3a, leading to their exclusion from the nucleus and subsequent transcriptional repression of apoptotic genes such as BCL2-like 11 (BIM), FASL, and PUMA [25]. AKT phosphorylates and inactivates GSK3β, hence stabilizing β-catenin and promoting cell cycle progression through cyclin D1. AKT additionally activates the mTORC1 complex, which facilitates protein synthesis and mitochondrial biogenesis, both essential for the proliferation of granulosa and cumulus cells [26]. These molecular processes collaboratively establish an anti-apoptotic and metabolically active follicular environment that facilitates the development of oocytes, particularly mature oocytes at metaphase II (MII) [27].

The exosomal transfer of miR-21 from somatic cells to neighboring cells, including the oocyte, enhances the survival of the cumulus–oocyte complex, suppresses follicular atresia, and fosters the cytoplasmic and nuclear competence of the oocyte. Increased levels of miR-21 in follicular fluid (FF) have been associated with enhanced MII oocyte yield, elevated fertilization rates, and improved blastocyst quality [28]. Jenabi et al. (2023) revealed that miR-21 expression in follicular fluid and cumulus cells was significantly elevated in patients yielding mature oocytes during ICSI cycles, correlating with enhanced IVF results [29].

The TGF-β signaling cascade, particularly the SMAD-dependent axis, governs follicular development, cumulus cell differentiation, and oocyte-somatic cell interaction. TGF-β family ligands, such as GDF9, BMP15, and activins, interact with type II and type I serine/threonine kinase receptors, leading to the phosphorylation of receptor-regulated SMADs (R-SMADs), including SMAD2/3 or SMAD1/5/8 [30]. R-SMADs subsequently assemble complexes with SMAD4 and translocate to the nucleus to modulate the transcription of genes associated with cumulus expansion, matrix remodeling, and meiotic resumption [31]. Among the exosomal miRNAs implicated in this mechanism, miR-92a and miR-130b have been identified as significant negative regulators inside human follicular fluid exosomes. Both miRNAs are primarily synthesized by granulosa and cumulus cells and exhibit differential expression in women with atypical ovarian response [32]. miR-92a targets SMAD5, a principal transducer in the BMP signaling pathway of the TGF-β superfamily, whereas miR-130b inhibits BMPR2, a type II receptor essential for BMP ligand-induced SMAD activation [33]. The downregulation of these targets hinders SMAD1/5 phosphorylation and obstructs the transcription of genes such as HAS2, TNFAIP6, and PTGS2, which are essential for hyaluronic acid synthesis, prostaglandin production, and cumulus matrix stability [34].

Functionally, these miRNA-mediated changes hinder cumulus expansion, diminish cumulus–oocyte metabolic coupling, and disturb the spatial organization of the oocyte microenvironment, eventually restricting the oocyte’s capacity to advance through meiosis and fertilization [35]. These repercussions may hinder the advancement of ooplasmic competence, chromatin remodeling, and cortical granule migration, all of which depend on robust cumulus cell signaling.

Apoptosis is a meticulously regulated process within the ovarian follicle essential for follicular selection, atresia, and the maintenance of granulosa and cumulus compartment homeostasis. Regulated apoptosis is essential for follicular remodeling; nevertheless, excessive or premature granulosa cell death can impair steroidogenesis, nutrient transport, and oocyte maturation [36]. Exosomal miRNAs, including miR-155-5p and miR-15a-5p, have been identified as crucial regulators of this balance, affecting both intrinsic (mitochondrial) and extrinsic (death receptor) apoptotic pathways in a cell-type-specific manner [37].

MiR-15a-5p is predominantly recognized as pro-apoptotic and is often shown to be raised in the exosomal content of poor ovarian responders. This miRNA targets the BCL2 gene, which encodes an anti-apoptotic protein that stabilizes mitochondrial membranes and inhibits cytochrome C release [38]. MiR-15a-5p inhibits BCL2, leading to mitochondrial outer membrane permeabilization, activation of caspase-9, and the progression of the intrinsic apoptotic pathway. Increased levels of exosomal miR-15a-5p in the follicles of women with diminished ovarian reserve or advanced maternal age lead to granulosa cell depletion, reduced estrogen production, and disrupted metabolic interaction with the oocyte [39]. These consequences are particularly detrimental in controlled ovarian stimulation techniques, as premature follicular atresia diminishes the quantity of retrievable MII oocytes.

Zhang et al. (2017) demonstrated that miR-15a-5p is significantly enhanced in follicular fluid exosomes of low responders during in vitro fertilization, correlating with diminished oocyte yield and suboptimal embryo development. These findings substantiate the notion that exosomal miRNAs not only mirror the apoptotic condition of the follicle but also actively modulate it through targeted post-transcriptional regulation of genes associated with survival. miR-155-5p and miR-15a-5p exemplify opposing mechanisms of apoptotic regulation within the follicular niche: the former safeguards follicular integrity, whilst the latter facilitates cell death and follicular degeneration in response to stress or aging. Their equilibrium inside exosomes may serve as a molecular indicator for follicular health, with potential use in diagnostic screening and therapeutic modulation in ART regimens aimed at enhancing oocyte quantity and quality [40].

The achievement of oocyte competence necessitates metabolic collaboration between cumulus cells and the oocyte, which is a physiologically semi-quiescent cell that predominantly depends on somatic support for energy substrates, chiefly pyruvate and lactate [35]. Cumulus cells take up glucose and utilize glycolysis to convert it into intermediates, which are subsequently delivered to the oocyte through transzonal projections and gap junctions. The somatic-germline metabolic connection is meticulously regulated by numerous signaling pathways, with new findings indicating that exosomal microRNAs, especially miR-143-3p, serve as potent post-transcriptional regulators of this relationship [41].

miR-143-3p is a short non-coding RNA that significantly influences metabolic regulation, adipogenesis, and insulin signaling. Exosomes are released by granulosa and cumulus cells within the ovarian follicle, and their concentration has been found to be significantly elevated in the follicular fluid of women with PCOS [42]. MiR-143-3p specifically targets the mRNAs encoding hexokinase 2 (HK2) and phosphofructokinase muscle type (PFKM), which are two rate-limiting enzymes in the glycolytic process. HK2 facilitates the phosphorylation of glucose to glucose-6-phosphate, so initiating glycolysis, while PFKM governs the commitment step that transforms fructose-6-phosphate into fructose-1,6-bisphosphate. The suppression of these enzymes diminishes glycolytic efficiency and restricts the production of pyruvate, lactate, and NADH [43]. The metabolic deficiency directly affects the oocyte, as pyruvate generated by glycolysis in cumulus cells is transported into the oocyte, where it is utilized in the tricarboxylic acid (TCA) cycle to produce ATP, sustain mitochondrial membrane potential, and facilitate meiotic spindle production. Insufficient availability of these substrates results in mitochondrial dysfunction, heightened oxidative stress, and inability to complete the meiotic cycle. Moreover, diminished NADH levels disturb redox balance, leading to the accumulation of reactive oxygen species (ROS) and subsequent harm to oocyte cytoplasmic constituents, including mitochondrial DNA and spindle microtubules.

The timely advancement of the cell cycle in granulosa and cumulus cells is essential for effective folliculogenesis, estrogen synthesis, and the provision of sufficient metabolic and structural support to the developing oocyte. Throughout follicular development, granulosa cells undergo many cycles of proliferation in response to FSH and growth hormones, facilitating follicular expansion and the formation of the cumulus–oocyte complex. The dysregulation of proliferative activity can affect oocyte development, meiotic maturation, and embryo competence. Among the exosomal microRNAs that regulate cell cycle dynamics, miR-424-5p has been identified as a potent suppressor of the cell cycle, especially in relation to PCOS and diminished ovarian function [44].

The reproductive age induces significant changes in the cellular and molecular structure of the ovarian follicle, especially in granulosa cells and the oocyte microenvironment. A notable indicator of ovarian aging is the alteration in the content and signaling capabilities of exosomes within the follicular fluid. Women with diminished ovarian reserve exhibit changes in the expression profiles of certain miRNAs that influence follicle viability, oocyte quality, and gonadotropin responsiveness, and a reduced number of exosomes. Hooten et al. (2013) identified more than 200 miRNAs in human follicular fluid exosomes, several of which exhibited differential expression with advancing age. Significantly, miR-320a, a transcriptional repressor of glycolytic and mitochondrial genes, was observed to be markedly downregulated in older women. The absence of miR-320a may lead to the overexpression of metabolic enzyme inhibitors, diminishing the energy capability of cumulus cells and compromising oocyte maturation. MiR-19b, an anti-apoptotic microRNA, was correspondingly diminished, leading to heightened expression of pro-apoptotic regulators like TP53INP1 and CASP7 in aged follicles [45].

Shen et al. (2023) conducted a targeted miRNA sequencing analysis in exosomes derived from the follicular fluid of women with clinically diagnosed diminished ovarian reserve (DOR) and identified a consistent upregulation of miR-23a, miR-34a, and miR-181a, all of which participate in DNA damage response, chromatin remodeling, and apoptosis induction. For instance, miR-34a directly targets SIRT1, a histone deacetylase crucial for safeguarding oocytes against oxidative stress and mitochondrial dysfunction. SIRT1 deficiency may lead to chromosomal misalignment, spindle abnormalities, and aneuploidy, commonly observed in aged oocytes. Moreover, miR-181a suppresses FOXO1 and ATM, two essential sensors of oxidative and genotoxic stress, hence diminishing the follicle’s capacity to respond adaptively to physiological damage. These age-related miRNAs disrupt normal oocyte somatic support, promote follicular atresia, diminish gonadotropin response efficacy, and lead to reduced MII oocyte yield and suboptimal embryo development in ART settings. These findings confirm the notion that exosomal miRNAs function as indicators of ovarian aging, conveying cumulative molecular damage signals within the follicular environment. Their dysregulation leads to abnormal gene expression patterns in granulosa and cumulus cells, accelerating the decline of follicular health. The relative abundance or depletion of specific miRNAs in exosomes may serve as a non-invasive diagnostic instrument for assessing oocyte quality, ovarian reserve status, and customized ovarian stimulation methods [46].

Table 1 shows that an expanding corpus of literature has recognized multiple ex-miRs as important regulators of the follicular milieu. These diminutive RNAs, encapsulated in extracellular vesicles released by granulosa cells and cumulus cells, modulate critical pathways such as PI3K–AKT, ERK1/2, glycolysis, and apoptosis. Table 1 illustrates that miR-21 facilitates CC survival and oocyte maturation by PTEN suppression and PI3K–AKT activation, whereas miR-34c improves blastocyst quality by targeting apoptotic and epigenetic regulators. In contrast, miRNAs including miR-15a-5p and miR-143-3p exert harmful effects, leading to follicular dysfunction in POR and PCOS. This functional diversity emphasizes the significance of characterizing ex-miR expression to forecast oocyte quality and inform treatment strategies during ARTs.

**Figure 1:**
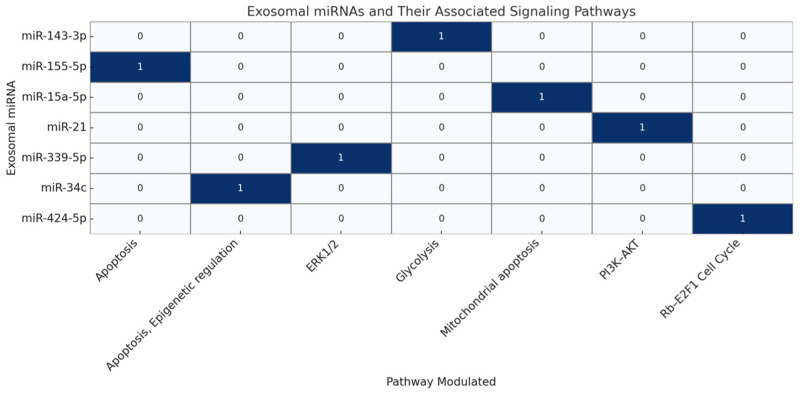
Exosomal microRNAs and Their Associated Signaling Pathways in the Follicular Environment.

Figure 1 exosomal microRNAs in the follicular environment together with their signaling pathways. Every cell in the matrix shows if a given ex-miR has been experimentally connected to the modulation of a given signaling pathway. Published research confirms or suggests an interaction; “1” denotes such a confirmed or hypothesized interaction; “0” denotes no known association. Figure 1 shows the heat map illustrating how important ex-miRs discovered in FF correlate with the signaling pathways they influence in GCs and CCs. Every cell in the matrix indicates if a given ex-miR has been experimentally connected to the modulation of a given pathway, including PI3K–AKT, ERK1/2, death, glycolysis, and Rb–E2F1 cell cycle control. Emphasizing their functional diversity and potential diagnostic uses in ARTs, the map presents a brief overview of how many ex-miRs control follicular development and oocyte maturation.

### 2.3. Roles of Cumulus Cells, Granulosa Cells, and the Oocyte in Exosomal Communication

Coordinated paracrine and exosomal interactions between granulosa and cumulus cells facilitate optimal oocyte development. This cross-talk is mediated not only by soluble substances and direct cellular interactions, but also by the bidirectional exchange of exosomes.

These vesicles facilitate communication among follicular cells. Each cell type in the follicular unit makes a unique contribution to the exosomal network, both as a transmitter and recipient, with cell-type-specific characteristics in exosomal biogenesis machinery, cargo composition, and uptake processes.

Exosomal microRNAs (miRNAs) present in the ovarian follicular environment are acknowledged as significant regulators of gene expression and intracellular signaling pathways that govern follicular development, somatic cell functionality, and oocyte competence. These miRNAs, contained within extracellular vesicles (EVs), are released by granulosa cells, cumulus cells, and even the oocyte itself [51]. They are conveyed to adjacent cells through active vesicular uptake processes, including receptor-mediated endocytosis and membrane fusion. Exosomal miRNAs associate with Argonaute proteins to form RISCs, which then attach to complementary sequences in target mRNAs, leading to translational suppression or mRNA degradation [52]. This technique enables accurate modulation of essential genes associated with oocyte maturation, cumulus expansion, and follicular integrity. Exosomal miRNAs have a significant role in human reproduction, as research demonstrates correlations between their expression in follicular fluid and several IVF outcomes, including oocyte maturity, fertilization rates, embryo quality, and clinical pregnancy rates [53].

The PI3K/AKT signaling cascade, governed by exosomal miRNAs, is crucial for the survival, proliferation, and metabolic flexibility of follicular cells. This system serves as a primary regulatory center, converting extracellular signals such as hormones and growth factors into intracellular responses that facilitate follicle development and oocyte maturation [54]. MiR-21, a microRNA continuously elevated in exosomes originating from follicular fluid, is mostly synthesized by cumulus cells in response to ovulatory triggers such as LH surge and FSH exposure [55]. MiR-21 directly interacts with the 3′ UTR of PTEN mRNA, a crucial phosphatase that suppresses the PI3K/AKT pathway through the dephosphorylation of PIP3. The inhibition of PTEN leads to the accumulation of PIP3 at the plasma membrane, facilitating the recruitment and phosphorylation of AKT at Ser473 and Thr308 by mTORC2 and PDK1, respectively [56]. Activated AKT phosphorylates various downstream targets, including FOXO transcription factors, BAD, GSK3β, and the mTORC1 complex, to propagate survival signals.

AKT phosphorylates FOXO1 and FOXO3, resulting in their exclusion from the nucleus and subsequent inactivation, which downregulates apoptosis-related genes such as BIM, PUMA, and FasL. AKT-mediated inactivation of GSK3β stabilizes β-catenin and cyclin D1, enhancing the proliferation of granulosa and cumulus cells. AKT promotes mTORC1, enhancing anabolic metabolism through increased protein synthesis, mitochondrial biogenesis, and glycolytic capacity, all essential for sustaining the developing oocyte [57]. This cascade enhances the cytoplasmic and nuclear maturation of the oocyte by improving nutrient availability, mitigating oxidative stress, and diminishing follicular atresia.

The miR-21-PTEN-PI3K/AKT axis functionally regulates both the somatic and germ cell compartments of the follicle. In the somatic compartment, it promotes cumulus expansion, extracellular matrix remodeling, and granulosa cell viability. In the germline compartment, it maintains the oocyte in a metabolic and anti-apoptotic environment that facilitates successful fertilization and early embryonic development. The significance of this pathway has been confirmed in human-assisted reproductive technologies. MicroRNAs such as miR-92a and miR-130b modulate the TGF-β/SMAD signaling pathway, affecting follicle development and embryo quality. This route is essential for cumulus expansion and interaction between the oocyte and somatic cells. miR-92a and miR-130b have been identified in follicular fluid exosomes and shown to target SMAD5 and BMPR2, respectively. The downregulation of these proteins inhibits the traditional SMAD2/3 and SMAD1/5/8 pathways, hence diminishing TGF-β signaling. This leads to diminished expression of cumulus expansion markers, including HAS2, PTGS2, and TNFAIP6, hence impairing extracellular matrix formation and the synchronized development of the cumulus–oocyte complex [58]. Correspondingly, miR-15a-5p is upregulated in exosomes from poor ovarian responders and targets BCL2, a critical anti-apoptotic protein. By decreasing BCL2, miR-15a-5p facilitates mitochondrial membrane permeabilization and cytochrome c release, hence triggering the intrinsic apoptotic pathway and endangering follicular viability. Zhang et al. (2017) reported an elevation of this miRNA in low-responder individuals undergoing ART [40].

Exosomal miRNAs modulate metabolic activity to maintain the oocyte’s energy equilibrium and metabolic proficiency. The cumulus–oocyte complex is significantly dependent on pyruvate and lactate generated from glycolysis, supplied by cumulus cells. miR-143-3p, identified as elevated in follicular fluid exosomes from patients with PCOS, directly targets HK2 and PFKM, two rate-limiting enzymes in glycolysis. Inhibiting these enzymes diminishes glucose flow through the glycolytic pathway, leading to reduced ATP and NADH production. The resultant metabolic deficiency undermines cumulus cell functionality and restricts the metabolic substrates available to the oocyte, hence impairing mitochondrial function, meiotic spindle formation, and cytoplasmic maturation. Cao et al. (2022) found that the dysregulation of miR-143-3p in PCOS impairs cumulus–oocyte metabolic coupling, leading to oocyte incompetence [48].

MiRNAs derived from exosomes also affect cell cycle regulation, especially in the granulosa and cumulus compartments. miR-424-5p, highly expressed in exosomes from PCOS follicles, inhibits critical cell cycle regulators including cyclin D1 (CCND1) and E2F1, both essential for the G1/S transition. Moreover, miR-424-5p targets CDCA4, a transcriptional regulator implicated in cell cycle entry. Discovered that elevated levels of exosomal miR-424-5p diminished CDCA4 expression, leading to reduced granulosa cell proliferation and follicular developmental stasis. The reduction in somatic support impedes cumulus expansion and disrupts the oocyte-somatic interactions necessary for competence acquisition. The exosomal miRNA profile undergoes significant alterations with advancing reproductive age and diminished ovarian reserve (DOR) [49]. Hooten et al. (2013) analyzed more than 200 miRNAs in human follicular fluid exosomes and identified age-related alterations in expression patterns, including reductions in miR-320a and miR-19b, alongside elevated levels of pro-apoptotic miRNAs. These mutations are deemed to exacerbate oxidative stress, mitochondrial dysfunction, and transcriptomic instability in both somatic and germ cells [45]. Shen et al. (2023) expanded upon these findings by illustrating that in patients with impaired ovarian reserve (DOR), atypical exosomal miRNA expression profiles were associated with suboptimal oocyte quality, reduced response to stimulation, and decreased implantation potential [46].

For example, miR-143-3p, which is typically overexpressed in PCOS patients’ exosomes, inhibits the expression of hexokinase 2 (HK2) and phosphofructokinase (PFKM) in cumulus cells, affecting glycolysis and limiting pyruvate availability to the oocyte [48]. This disturbed energy exchange markedly hinders oocyte mitochondrial function and meiotic maturation. Likewise, miR-378a, a notable exosomal miRNA produced from MGC, suppresses CYP19A1 (aromatase), resulting in reduced estradiol production and a decrease in the oocyte’s estrogenic support—an effect that is particularly significant in conditions of diminished ovarian reserve and hyperandrogenism.

MGC exosomes encompass proteins that regulate oxidative stress, including peroxiredoxins (PRDXs), heat shock proteins (HSP70, HSP90), and glutathione S-transferases (GSTs), which aid in safeguarding the follicle from ROS-induced injury during fast follicular expansion. Moreover, their cargo comprises signaling peptides and transcriptional regulators that affect FSHR responsiveness and facilitate estradiol-dependent gene transcription in adjacent cells. In pathological conditions like PCOS, the exosomal output of MGCs alters in terms of miRNA composition, exosome size, concentration, and targeting selectivity.

MGC-derived exosomes containing higher amounts of miR-424-5p decrease CDCA4 and cyclin D1, therefore reducing mural granulosa formation and follicle enlargement. Furthermore, their ability to transfer pro-angiogenic factors such as VEGFA mRNA fragments and miR-126 is reduced, presumably resulting in inadequate follicular vascularization and luteinization [49].

Historically, the oocyte was thought to be a mostly passive recipient of somatic support within the ovarian follicle, relying on cumulus and granulosa cells for metabolites, growth factors, and meiotic control. However, recent evidence suggests that the oocyte may play a more active role in exosome-based communication, both by uptake of exogenous vesicles and possibly by production of its own small extracellular vesicles (sEVs) [59]. Although direct isolation and profiling of oocyte-derived exosomes in vivo remains technically challenging in humans, animal models, and in vitro maturation systems have shown that maturing oocytes can release vesicles enriched in regulatory non-coding RNAs like miR-27b and miR-10a, as well as lncRNAs like NEAT1 and H19 [21].

In parallel, the oocyte has a highly specific vesicle uptake mechanism. It expresses phosphatidylserine receptors (such as TIM-4), heparan sulfate proteoglycans, and scavenger receptors, which aid in the internalization of exosomes originating from cumulus and granulosa cells.

Exosomal contents, particularly miRNAs (e.g., miR-21, miR-146a, miR-132), long non-coding RNAs (e.g., GAS5), and protein complexes, enter the cytoplasm and regulate mitochondrial dynamics, redox balance, mRNA translation, and epigenetic remodeling [60].

Exosomal signaling plays a vital role in cytoplasmic maturation, which involves organelle translocation, ATP enrichment, and the stability of maternal mRNA. For example, miR-21-enriched exosomes produced from cumulus cells have been found to boost AKT activation in the oocyte, resulting in decreased FOXO3A activity and greater resistance to apoptosis—a crucial trait of competent MII oocytes [29].

Similarly, exosomal lncRNA H19 has been linked to EZH2-dependent chromatin remodeling, which facilitates the silencing of transcriptionally active regions during the GV to MII oocytes transition [61].

Furthermore, oocytes are temporally sensitive to exosomal input: their ability to internalize and respond to exosomal signals appears to peak during the periovulatory window, when gap junction closure restricts direct transzonal exchange and exosome-mediated signaling becomes more prevalent. This phase overlaps with the peak exosomal secretion activity from cumulus and mural granulosa cells, showing a coordinated regulatory network focused on final oocyte maturation. Pathological diseases such as PCOS and advanced maternal age can impair the oocyte’s ability to accept and comprehend exosomal cues. Impaired exosome uptake has been linked to abnormal oolemma composition, altered expression of endocytosis-related genes (e.g., caveolins, flotillins), and increased oxidative stress, which could explain some of the deficiencies in oocyte competence found in these populations [45].

### 2.4. Altered Exosomal Signaling in PCOS and DOR

Disruptions in the precise communication between oocytes and adjacent somatic cells are characteristic of reproductive disorders such as PCOS and DOR. Recent evidence indicates that exosomal signaling is crucial for facilitating intercellular communication, and that changes in exosomal cargo—such as miRNAs, lncRNAs, proteins, and metabolites—are implicated in follicular dysfunction, compromised oocyte maturation, and suboptimal outcomes in ARTs.

#### 2.4.1. Exosomal miRNA Dysregulation in PCOS: Senescence, Metabolic Blockade, and Apoptosis

PCOS is characterized by anovulation, hyperandrogenism, and abnormal folliculogenesis, and has been increasingly linked to cellular miscommunication inside the ovarian follicle. miRNAs, released into the follicular fluid, act as powerful mediators of intercellular signaling. Growing data in PCOS suggests that specific miRNAs are dysregulated in exosomes from granulosa cells, resulting in follicular dysplasia, apoptosis, and oocyte incompetence.

Yuan et al. (2021) discovered, among other results, a downregulation of miR-424-5p in exosomes produced from follicular fluid in patients with PCOS. MiR-424-5p directly targets the transcription factor CDCA4, modulating the Rb/E2F1 pathway, which is crucial for cellular proliferation and senescence [49]. Reduced levels of miR-424-5p result in the overexpression of CDCA4, which hastens granulosa cell senescence, inhibits mitotic activity, and decreases proliferative support to the oocyte. Exosomes deficient in miR-424-5p, when administered to granulosa cells, demonstrated enhanced β-galactosidase staining, reduced proliferation indicators, and the activation of senescence-associated pathways [62]. The functional implications are significant when preantral or small antral stage follicular development halts, hence affecting dominant follicle selection and leading to ovulation failure [49].

Cao et al. (2022) [48] concurrently elucidated the dysregulation of glycolysis-related miRNAs, specifically miR-143-3p and miR-155-5p, in exosomes derived from the follicular fluid of PCOS patients. While miR-143-3p was overexpressed, leading to an antagonistic imbalance in the regulation of metabolic and apoptotic processes, miR-155-5p was significantly downregulated. In vitro, miR-143-3p inhibited the expression of glycolytic enzymes HK2 and PKM2 in KGN cells (human granulosa-like tumor cells), thereby reducing glucose metabolism, lactate production, and ATP synthesis. The increased production of pro-apoptotic markers, such as BAX and Caspase-3, ensued from these metabolic deficiencies. Intriguingly, the forced overexpression of miR-155-5p reversed this effect in experimental conditions by reinstating glycolytic activity and enhancing granulosa cell viability. This suggests a context-dependent, potentially protective role for miR-155-5p in PCOS, presumably by mitigating the pro-apoptotic effects elicited by miR-143-3p.

Importantly, these miRNAs do not function in isolation. Essential components of miRNA regulation encompass functional redundancy and cooperation; the synergistic effects of miR-143-3p, miR-424-5p, and others in PCOS appear to coalesce around three critical nodes:

- Cell Cycle Arrest and Senescence Induced by CDCA4, p21, and p16INK4A
- Regulation of Metabolism through LDHA, HK2, and PKM2
- Regulation of Apoptosis by Caspases and BCL2 Family Proteins

These modifications collectively undermine the granulosa–oocyte support system, contributing to the manifestation of the PCOS subfertile phenotype. Their accessibility via minimally invasive methods is facilitated by their presence in follicular fluid exosomes, so categorizing them as potential diagnostic biomarkers and therapeutic targets for the restoration of follicular competency.

#### 2.4.2. Exosomal Molecular Alterations in Reproductive Senescence and DOR

The reduction in follicle count, along with a significant decline in oocyte quality and somatic support, indicates the decrease in female fertility with advancing age, especially in the pathological condition of diminished ovarian reserve (DOR) [63]. A growing body of data links this functional decline, largely driven by extracellular vesicles, particularly exosomes, to molecular instability within the ovarian milieu. These nanoscale vesicles function as sophisticated carriers for intercellular communication by delivering regulatory chemicals like as miRNAs, lncRNAs, proteins, and metabolites from somatic follicular cells to the oocyte. Alterations in gene expression, mitochondrial function, and epigenetic plasticity, coupled with modifications in the composition of exosomal cargo that reflect the internal biochemical irregularities of the follicle, substantially influence oocyte aging [64].

Employing GC–TOFMS, a significant work by Gu et al. (2024) examined the exosomal metabolome in follicular fluid from women of advanced reproductive age, uncovering age-related modifications in seventeen key compounds. These metabolites are involved in critical processes for oocyte competency, such as redox control (e.g., glutathione derivatives), amino acid catabolism (e.g., methionine, tryptophan), and lipid biosynthesis (e.g., cholesterol intermediates) [65]. These modifications signify a disturbance in mitochondrial coupling and granulosa cell metabolism. Reduced concentrations of pyruvate and lactate hinder the energy substrate availability for the oocyte, hence restricting the ATP necessary for spindle assembly, organelle localization, and the translational activation of maternal mRNAs [29]. The elevation of oxidative byproducts, as evidenced by disrupted glutathione metabolism, implies inadequate redox buffering, hence subjecting the oocyte to DNA damage and mitochondrial depolarization induced by reactive oxygen species (ROS) [65].

Sang et al. (2013) discovered more than 200 exosomal miRNAs in human follicular fluid at the transcriptome level, observing significant age-related changes [66]. Older women’s FF consistently downregulated miRNAs linked to DNA methylation and chromatin maintenance, such as miR-320a, miR-19b, and let-7 [67]. The suppression of these miRNAs can interfere with the control of DNMT1, EZH2, and HDAC2, leading to the loss of transcriptional repression, chromatin decondensation, and abnormal gene expression in oocytes and cumulus cells [68]. This condition affects chromatin condensation during meiosis, increasing spindle instability and aneuploidy, which are major factors in implantation failure and early miscarriage in older persons. Concurrently, increased concentrations of miRNAs, such as miR-34a and miR-21, are acknowledged as triggers of cellular apoptosis. Their heightened expression probably promotes the transcription of pro-apoptotic genes such as BAX, PUMA, and CASP9, especially during oxidative stress when mitochondrial membrane integrity is compromised [66].

Age-associated systemic inflammatory changes intensify these transcriptome abnormalities. Hooten et al. (2013) established that three miRNAs—MiR-181a-5p, miR-151a-5p, and miR-1248—present in FF-derived exosomes were correlated with a systemic reduction in blood levels. miR-181a enhances the expression of anti-inflammatory cytokines, including TGF-β and IL-10, while inhibiting IL-6 and TNFα. The inhibition of this pathway may lead the follicular milieu to sustained inflammation, intensifying DNA damage, and threatening the immunological privilege of the follicle. The observations support the notion that the age-associated decline in exosomal anti-inflammatory signals may expedite granulosa cell senescence and hinder follicular reorganization [45].

These alterations yield an exosomal cargo profile that precludes both autonomous oocyte repair mechanisms and cooperation between granulosa cells and oocytes. The reduced transfer of electron transport cofactors and translational regulators compromises mitochondrial activity. Altered miRNA regulation causes dysregulation of histone-modifying enzyme levels, leading to asynchrony between nuclear and cytoplasmic maturation. The buildup of reactive oxygen species compromises spindle checkpoints and hastens telomere degradation. Despite ultrasonic imaging indicating physically normal follicles, these converging circuits render the egg incapable of fertilization or subsequent embryonic development.

#### 2.4.3. Exosomal Regulation of Aging and Impaired Follicular Intracellular Signaling Pathways

The preservation of oocyte developmental potential is significantly influenced by age-sensitive interactions between somatic and germline elements within the follicle, facilitated via signaling vesicles and metabolic exchanges. Exosomes discharged into the follicular fluid function as signaling agents that transmit regulatory RNAs, proteins, and metabolic signals, thus influencing the immediate cellular microenvironment [69].

These vesicles, serving as molecular messengers, affect oocyte competence, metabolic balance, chromatin architecture, and cellular fate determinations. This signaling network is predominantly affected by age-related changes in exosomal cargo concerning reproductive aging and diminished ovarian reserve (DOR) [70]. These alterations substantially impact intracellular pathways, including PI3K/AKT, MAPK/ERK1/2, TGF-β/SMAD, AMPK, and epigenetic processes, hence compromising follicular function and oocyte developmental potential.

Exosomal miRNAs in aged follicles influence a pivotal pathway related to the PI3K/AKT/PTEN axis. Under typical conditions, FSH and growth hormones stimulate PI3K, which phosphorylates AKT, hence enhancing anabolic processes, proliferation, and anti-apoptotic signaling in granulosa cells [71]. PTEN, a tumor suppressor, acts as a negative regulator by preventing the overactivation of pathways. In the process of aging, exosomal miR-21, secreted by neighboring cumulus cells, is markedly increased and specifically targets the 3′ UTR of PTEN mRNA, leading to its destruction [72]. This leads to extended PI3K activation and AKT phosphorylation. While this may initially enhance cell survival, prolonged activation results in increased mTORC1 signaling, hence inhibiting autophagy and facilitating the buildup of damaged organelles and misfolded proteins. Overactivated AKT further inactivates FOXO3a, a crucial transcription factor involved in antioxidant defense (e.g., catalase, SOD2) and DNA repair, thereby shifting the balance towards oxidative stress, GC senescence, and reduced oocyte support [73].

The modified exosomal composition is accompanied by poor MAPK/ERK1/2 signaling, which governs transcriptional responses to hormone signals and coordinates oocyte development. Increased concentrations of miR-339-5p in follicular fluid exosomes target SFPQ, an RNA-binding protein that modulates ERK1/2 activation, as documented by Han et al. (2024) [50]. The suppression of SFPQ reduces ERK1/2 phosphorylation, hence impairing the transcriptional activation of downstream targets including HAS2, PTGS2, and TNFAIP6, which are critical for meiotic development and cumulus matrix synthesis [74]. This cascade is crucial for cumulus growth, spindle positioning regulation, mitochondrial redistribution, and cortical granule polarization. Thus, the lack of ERK1/2 activation results in insufficient cytoplasmic maturation of oocytes, lower in vitro fertilization success rates, and decreased frequencies of blastocyst development.

The TGF-β/SMAD pathway is a crucial signaling axis influenced by exosomal miRNAs. miR-92a and miR-130b in follicular fluid exosomes from aged follicles, targeting two critical components of the canonical SMAD1/5/8 pathway. Reducing the phosphorylation of these SMADs allows these miRNAs to suppress the transcription of genes related to GC proliferation, matrix remodeling, and oocyte-somal synchronization. This cascade is crucial for transducing oocyte-secreted ligands, including GDF9 and BMP15; therefore, its inhibition undermines the paracrine loop necessary for follicular integrity. Furthermore, the alteration in TGF-β signaling affects ID proteins and downstream anti-apoptotic molecules, hence making GCs vulnerable to stress-induced apoptosis [75].

From a metabolic standpoint, exosomal anomalies affect the AMPK energy-sensing system. Gu et al. (2024) report that exosomes from the follicular fluid of older women have reduced concentrations of pyruvate, lactate, and NAD^+^, which are essential activators of AMPK. Without AMPK activation, the oxidative stress response mechanisms controlled by SIRT1, along with oocytes and neighboring granulosa cells, cannot commence PGC1α-induced mitochondrial biogenesis. Mitochondrial dysfunction is marked by reduced membrane potential, increased reactive oxygen species (ROS), and an ATP deficit. These characteristics cause meiotic arrest or aneuploidy by hindering the egg’s cytoplasmic maturation, blocking meiotic processes, and consequently reducing the capacity for normal spindle assembly and chromosomal alignment [65].

Exosomes linked to aging simultaneously influence epigenetic remodeling, an essential phase in oogenesis. Exosomes from aged individuals display reduced concentrations of miRNAs, including miR-320a and miR-19b, which modulate chromatin-modifying enzymes like DNMT3A, EZH2, and HDAC1, as documented by Sang et al. (2013). Their downregulation may result in inaccurate histone modifications, diminished de novo methylation, and an incapacity to silence genes that ought to stay dormant during meiosis. The resultant effects are transcriptional noise, unstable chromatin structure, and inadequate imprinting. Following conception, epigenetic abnormalities, including abnormal histone alterations, reduced DNA methylation, and the loss of transcriptional repression, may jeopardize chromatin integrity and increase the likelihood of embryonic developmental failure [66].

These signaling axes operate as an integrated network rather than independently. The reduction in ERK1/2 activity is associated with decreased SMAD signaling, obstructing the cumulus expansion program, whereas sustained overactivation of PI3K/AKT reduces AMPK responsiveness. Likewise, the increase in ROS-induced DNA breaks, which cannot be sufficiently repaired in the absence of FOXO3a and SIRT1 signaling, leads to heightened chromatin instability due to mitochondrial failure.

## 3. Exosomal microRNAs and Oocyte Maturity in In Vitro Fertilization

The attainment of oocyte maturity is a meticulously regulated process that entails coordinated molecular, metabolic, and epigenetic signaling between the oocyte and its adjacent cumulus and granulosa cells [76]. This complex interaction is primarily facilitated by exosomal miRNAs found in FF, which serve as crucial regulators of gene expression within cellular compartments.

These diminutive non-coding RNAs, encapsulated in EVs, traverse the follicular fluid milieu, affecting processes like meiosis restart, cytoplasmic maturation, metabolic adaptation, and chromatin architecture. Within the realm of ARTs, the profile of exosomal miRNAs has proven to be a significant predictor of oocyte competence, fertilization capability, and blastocyst development [77].

### 3.1. Molecular Instruments for MII Oocytes Development

The attainment of MII oocyte competence is a meticulously orchestrated process regulated by specific nuclear and cytoplasmic maturation programs, driven by signaling pathways and transcriptional regulators inside the follicular niche [78]. Ex-miRs originating from FF-EVs are essential in this process, serving as intercellular messengers that convey gene-regulating material from GCs and CCs to the oocyte and neighboring somatic cells. By attaching to complementary regions in the 3′ UTRs of target mRNAs, these miRNAs induce post-transcriptional effects that lead to either translational repression or destruction through the miRNA–RISC complex. During the final phases of folliculogenesis, the stability and specificity of these miRNAs, enhanced by their exosomal encapsulation, make them powerful regulators of follicular development and oocyte maturation.

The research conducted by Santonocito et al. (2014) discovered 32 ex-miRs that were considerably enriched compared to plasma from the same subjects, thereby offering the first systematic characterization of human FF-EVs. These miRNAs targeted mRNAs associated with the inhibition of meiotic re-entry, including CDKN1A, a cyclin-dependent kinase inhibitor that binds and inactivates the CDK1–CCNB1 complex (MPF), BTG2, which suppresses cyclin expression and mRNA stability, and FOXO3, a forkhead transcription factor that promotes the expression of apoptotic and cell cycle arrest genes. The ex-miRs reduce the inhibitory limitation on meiotic development by downregulating these components, thereby promoting the activation of MPF, GVBD, and the consequent assembly of the metaphase spindle. Moreover, specific discovered miRNAs that govern epigenetic modulators, including HDAC2 and DNMT3A, further enhance the chromatin remodeling crucial for MII oocyte competency [11].

Han et al. (2024) clarified the mechanical function of miR-339-5p by the use of porcine COC models. MiR-339-5p directly inhibits SFPQ, a nuclear RNA-binding protein involved in the transcriptional control of genes downstream of the ERK1/2 pathway. The promoter regions of ELK1 and c-MYC, transcription factors critical for cumulus expansion and metabolic activity, are known to interact with SFPQ. The suppression of SFPQ by miR-339-5p resulted in decreased phosphorylation of ERK1/2 (p-ERK1/2), altered expression of cumulus-specific extracellular matrix genes (HAS2, PTGS2, TNFAIP6), and disrupted cytoskeletal reorganization and cortical granule translocation, which are suggestive of oocyte maturity. These alterations led to a reduced yield of MII oocytes and a lower blastocyst formation rate. Moreover, the overexpression of miR-339-5p modified ER stress indicators and lipid metabolism, hence causing ooplasmic abnormalities that compromise developmental competence. The inhibition of the SFPQ–ERK1/2 pathway by ex-miR undermines both the post-translational and transcriptional mechanisms essential for the acquisition of MII oocytes [50].

In 2024, Benedetti et al. found that oocyte competency was predominantly affected by miR-34c. Increased expression of miR-34c in FF-EVs was associated, as evidenced by miRNA sequencing and functional validation assays, with oocytes reaching the BL stage during in vitro fertilization. Among several targets, miR-34c specifically targets BCL2, an anti-apoptotic regulator of the mitochondrial membrane, and SIRT1, a NAD+-dependent deacetylase that modulates chromatin structure, mitochondrial biogenesis, and responses to oxidative stress. The downregulation of SIRT1 by miR-34c leads to hyperacetylation of histones and chromatin condensation, hence promoting effective meiotic spindle assembly. Moreover, the reduction in BCL2 balances the regulated apoptotic pruning necessary for optimum oocyte maturation with pro-survival signals. The inhibition of miR-34c resulted in mitochondrial depolarization and insufficient growth, while the administration of miR-34c mimics during in vitro maturation (IVM) raised egg cleavage rates and blastocyst quality, as demonstrated by an increased cell count and improved morphology [47].

Gu et al. (2024) advanced the comprehension of MII oocyte competency from a metabolic standpoint using GC–TOFMS analysis of FF-EV metabolites in young and AMA women [65]. Citrate, lactate, and cholesterol esters, essential substrates for mitochondrial respiration, histone acetylation, and steroidogenesis, exhibited variable abundance in metabolites. Enzymes modulated by miRNA, including HK2, PFKM, and CYP19A1, regulate these metabolites upstream, indicating that variations in ex-miR concentration in FF-EVs influence both signaling and the supply of nutrients and energy to the oocyte. In people with advanced maternal age (AMA) and diminished ovarian reserve (DOR), the reduction in NADH-generating substrates, along with decreased tricarboxylic acid (TCA) cycle activity, may result in insufficient ATP synthesis, elevated reactive oxygen species (ROS), and impaired spindle integrity [65].

### 3.2. Predictive Significance of Exosomal MicroRNAs Derived from Follicular Fluid-Based Assisted Reproductive Technology Outcomes

Physicians and researchers are exploring molecular markers to forecast the developmental potential of oocytes in assisted reproductive technology, with ex-miRs found in follicular fluid extracellular vesicles receiving heightened interest as diagnostic and prognostic tools. Unlike invasive biopsy techniques, the study of extracellular vesicle cargo offers a real-time assessment of the molecular processes involved in oocyte maturation by non-destructive exploration of the follicular environment. Ex-miRs, emitted by actively communicating cells such as CCs, GCs, and the oocyte, gather and transmit precisely regulated signals that reflect the functional condition of the follicle. Essential for folliculogenesis and the oocyte’s progression to developmental competence, their regulatory role encompasses PI3K/AKT, MAPK, TGF-β, and mTOR across several pathways. Moreover, their remarkable stability in biological fluids, attributed to encapsulation in lipid bilayers, makes them optimal candidates for biomarkers appropriate for integration into IVF procedures.

Santonocito et al. (2014) laid the foundation for this application by discovering a panel of 32 ex-miRs enriched in FF-EVs from women undergoing ICSI, which were unique from the miRNAs present in the plasma of the same people. A bioinformatic analysis identified several ex-miRs, including CDKN1A, FOXO3, and BTG2, which target genes that hinder meiotic resumption and follicular development. The downregulation of these targets promoted the activation of the CDK1/CCNB1 complex, allowing advancement from GVBD to MII oocytes. The direct identification of miRNAs using an uncomplicated FF sample, which mirrors functional molecular activities, offers a non-invasive method to evaluate oocyte readiness prior to denudation or fertilization. Significantly, the same ex-miRs also modulated elements of the TGF-β pathway, such as SMAD7 and BAMBI, which function as antagonists to conventional SMAD2/3 signaling. Their inhibition enhances the expression of cumulus expansion genes and optimizes communication between cumulus cells and oocytes via gap junctions, hence improving oocyte maturation [11].

Han et al. (2024) focused on miR-339-5p in a porcine model, hence augmenting the therapeutic potential of ex-miR biomarkers. They established that decreased maturation and lower BL formation in FF-EVs correlated with increased levels of miR-339-5p. Mechanistically, miR-339-5p directly inhibits SFPQ, a co-regulator of ERK1/2 activity, hence reducing the phosphorylation of MAPK3/1. This disruption leads to reduced transcription of ECM remodeling genes, including HAS2 and PTGS2, which are crucial for cumulus growth and the release of meiotic inhibitory signals. Moreover, miR-339-5p modified mitochondrial lipid metabolism by enhancing lipid droplet accumulation and diminishing mitochondrial function indicators, such as ATP levels and mitochondrial membrane potential. Cytoskeletal instability, metabolic stress, and transcriptional silencing synergistically hindered the oocyte’s advancement to metaphase II, hence limiting embryonic development. Thus, measuring miR-339-5p in FF-EVs constitutes a reliable predictive approach for detecting poor follicles prior to retrieval [50].

Benedetti et al. (2024) identified that miR-34c in FF-EVs functions as a beneficial indicator for oocyte developmental potential. MiR-34c levels were markedly increased in bovine models originating from follicles producing competent oocytes that reached the blastocyst stage during IVF. Functional investigations have shown that the suppression of miR-34c resulted in reduced developmental outcomes, while the incorporation of miR-34c mimics into IVM media promoted blastocyst formation and increased total cell counts. MiR-34c functions at the molecular level by targeting SIRT1 and BCL2. The hyperacetylation of histones H3 and H4, along with SIRT1 suppression, promotes chromatin remodeling, hence establishing a transcriptionally favorable environment necessary for genes involved in oocyte development. During cytoplasmic maturation, the downregulation of BCL2 balances oxidative stress with survival by modulating the mitochondrial death threshold, thereby promoting metabolic optimization. The data clearly support the use of miR-34c as a predictive biomarker and a functional enhancer of oocyte competency, with possible therapeutic implications in in vitro maturation techniques [47].

The research conducted by Ducarre et al. (2024) showed that FF-EVs from patients with PCOS and DOR displayed modified miRNA cargo and unique physicochemical properties, including increased polydispersity and fluctuations in zeta potential. These modifications aligned with significant variations in exosome synthesis and cargo integration in the afflicted follicles [79]. FF-derived ex-miRs hold prognostic importance as they encompass the molecular markers indicative of oocyte competency, hormone responsiveness, and metabolic preparedness. These molecules rapidly translate into clinical outcomes, such as MII oocyte yield, fertilization success, and blastocyst development, illustrating the dynamic interactions among follicular components. In individuals with PCOS, DOR, or AMA, the utilization of multi-marker panels—including miR-34c, miR-21, miR-339-5p, miR-143-3p, and related metabolites—demonstrates promise for improving embryo selection, tailoring ovarian stimulation, and augmenting ART outcomes.

### 3.3. Functional Integration in Hormonal Signaling

The ovarian follicle is a unique milieu where endocrine and paracrine signals converge to facilitate oocyte maturation. Ex-miRs produced by FF-derived cells modulate transcriptional and post-transcriptional networks in hormone-responsive cells, rendering them essential mediators of this complex signaling network. Enclosed within extracellular vesicles, these ex-miRs navigate the follicular fluid and precisely target cumulus cells, mural granulosa cells, and the oocyte itself [80]. By modifying downstream signal transmission, they ensure accurate temporal regulation of essential processes such as meiotic resumption, steroidogenesis, and CC-oocyte metabolic collaboration, consequently enhancing hormonal signals, including FSH and LH.

Follicle-stimulating hormone (FSH) binds to its G protein-coupled receptor (FSHR) on the plasma membrane of granulosa cells, forming the basis of follicular endocrine regulation. This link increases intracellular cAMP levels, activates adenylate cyclase, hence increasing PKA and the consequent phosphorylation of CREB [81]. This transcription factor enhances E2 synthesis by interacting with CRE sites on the promoters of steroidogenic enzymes, including CYP19A1 (aromatase) and HSD17B1. PKA activation concurrently sustains GC proliferation and augments the expression of anti-apoptotic genes (e.g., BCL2, MCL1) [82]. The effectiveness of this system is largely dependent on the cellular metabolic state, particularly ATP availability, which is reduced in pathological follicles due to ex-miRs that inhibit glycolytic enzymes.

The LH-induced EGFR–MAPK3/1 (ERK1/2) pathway is of similar importance. Mural granulosa cells increase the expression of amphiregulin (AREG) and epiregulin (EREG) in response to the mid-cycle luteinizing hormone surge, therefore activating the epidermal growth factor receptor (EGFR) on cumulus cells. This cascade sequentially activates RAS, RAF, MEK1/2, and ultimately ERK1/2. Phosphorylated ERK1/2, an essential mediator of CC expansion, translocates to the nucleus, where it promotes the transcription of genes such as HAS2, PTGS2, and TNFAIP6—critical for breaking gap junctions and enabling the resumption of meiosis in oocytes. MiR-339-5p, prevalent in FF-EVs from defective follicles, is shown by Han et al. (2024) to suppress SFPQ, a splicing factor and transcriptional co-regulator crucial for ERK1/2-mediated gene activation. ERK1/2 cannot effectively activate its targets without sufficient SFPQ activity, leading to ineffective cumulative expansion and insufficient support for the nuclear maturation of the oocyte. MiR-339-5p diminishes p-ERK1/2 levels, hence hindering meiotic growth and cytoskeletal reorganization [50].

An additional degree of integration exists between miRNA regulation and ovarian steroidogenesis. LH not only modulates MAPK signaling but also triggers acute steroidogenesis via the activation of STAR and CYP11A1. STAR enables the translocation of cholesterol into mitochondria, where CYP11A1 catalyzes its conversion into pregnenolone, the precursor of progesterone (P4). MiR-34c, recognized as common in FF-EVs linked to competent oocytes, inhibits SIRT1, a NAD+-dependent deacetylase that modulates FOXO transcription factors and PGC1α. By downregulating SIRT1, miR-34c alleviates the control of STAR, hence enhancing progesterone synthesis and mitochondrial support for the oocyte. It also diminishes BCL2, hence increasing the vulnerability of cumulus cells to redox fluctuations that facilitate chromatin remodeling during the MII transition.

The TGF-β/BMP signaling axis is another endocrine–paracrine interaction influenced by ex-miRs. In granulosa cells and cumulus cells, oocyte-derived BMP15 and GDF9 collaborate with BMPR2 to activate SMAD1/5/8 [83]. This stimulates the transcription of anti-apoptotic, glycolytic, and cell growth-associated genes. miR-92a and miR-130b, diminish BMPR2 and SMAD5, hence regulating this pathway. Decreased CC propagation, inadequate mitochondrial transfer to the oocyte, and disruption of metabolic cooperativity are among the subsequent effects. Consequently, a primary method via which FF-EVs influence somatic and germ cell growth in response to hormone signals is the suppression of BMP signaling. Ex-miR activation exacerbates hormonal dysregulation in insulin-resistant states such as PCOS. Insulin often enhances FSH signaling through the PI3K–AKT pathway, hence promoting granulosa cell development and augmenting IGF1R sensitivity [84].

### 3.4. Exosomal miRNA Signatures in Polycystic Ovary Syndrome and Suboptimal Responders

In subfertile populations, such as those with PCOS and poor POR, the composition of follicular fluid-derived EVs and their miRNA content significantly diverges from physiological standards [79]. This molecular reprogramming actively contributes to the dysfunction observed in these clinical symptoms, rather than only reflecting the follicular state. The anomalous existence, concentration, and targeting patterns of specific ex-miRs in these populations significantly interfere with intracellular signaling networks in GCs and CCs, hence undermining meiotic and cytoplasmic maturation and metabolic support for the egg. These miRNAs orchestrate alterations in critical regulatory networks, including PI3K–AKT, MAPK, TGF-β, and apoptotic pathways, thereby establishing a follicular environment detrimental to developmental competence [85]. These ex-miRs demonstrate potential not only as therapeutic targets for the restoration of ovarian function but also as prospective non-invasive biomarkers for diagnosis and categorization in ARTs.

The miRNA profile of FF-EVs in POR individuals is characterized by a pathological elevation of pro-apoptotic molecules such as miR-15a-5p and a reduction in miRNAs linked with survival and proliferation, including miR-320a and miR-19b. MiR-15a-5p, discovered by Zhang et al. (2017), targets BCL2, a mitochondrial membrane-associated protein that diminishes cytochrome c release and caspase activation. Downregulation of BCL2 induces miR-15a-5p to trigger mitochondrial outer membrane permeabilization, resulting in the release of apoptogenic factors such as cytochrome c and AIF, thereby initiating the intrinsic apoptotic pathway [40]. The final activation of caspase-9 and caspase-3 leads to germ cell death, hence diminishing the somatic support network essential for oocyte nourishment and steroidogenesis. The simultaneous downregulation of miR-320a and miR-19b removes beneficial signals for cell survival pathways, such as β-catenin activation and AKT phosphorylation, from the follicle. The combined effects are follicular atresia, inadequate cumulus expansion, and oocyte arrest before the MII oocytes transition. Yazdanian et al. (2024) observe that this miRNA pattern is frequently observed in individuals with Diminished Ovarian Reserve (DOR), correlating clinically with a subdued response to Controlled Ovarian Stimulation (COS), inadequate oocyte retrieval rates, and diminished embryo developmental potential [86].

MiR-424-5p is pivotal in the pathophysiology of follicular arrest associated with PCOS. This miRNA is significantly downregulated in FF-EVs from PCOS patients, as demonstrated by Yuan et al. (2021), resulting in the disinhibition of its direct target, CDCA4. CDCA4 induces cell cycle exit and cellular senescence in GC by activating the Rb–E2F1 pathway. Senescent granulosa cells are characterized by diminished proliferation, altered morphology, reduced steroidogenic capacity, and inability to maintain gap junction connectivity with the oocyte. This results in developmental incompetence and premature follicular aging. The in vitro reintroduction of miR-424-5p was found to decrease CDCA4, enhance proliferative capacity, and reactivate mitotic and metabolic functions in GCs. These findings highlight miR-424-5p as a crucial regulatory element and a potential therapeutic target for mitigating GC aging in PCOS follicles [49].

In addition to energy metabolism and cell cycle regulation, ex-miRs in PCOS significantly regulate inflammatory and steroidogenic pathways. Typically diminished in PCOS FF-EVs, miR-21 and miR-93 are recognized as inhibitors of pro-inflammatory cytokines such as TNFα, IL-6, and components of the NLRP3 inflammasome [44]. Their downregulation induces persistent low-grade inflammation in the follicular niche, hence exacerbating insulin resistance, oxidative stress, and deleterious signaling. Moreover, diminished levels of miR-132—a recognized post-transcriptional inhibitor of CYP17A1—result in excessive androgen synthesis, hence exacerbating hyperandrogenemia and its detrimental effects on follicular growth [87]. This steroidogenic disorder results in FSHR downregulation and diminishes hormonal responsiveness, hence establishing a detrimental cycle of follicular dysfunction and anovulation.

Proteomic and metabolomic analyses of FF-EVs enhance comprehension of the structural and metabolic consequences of miRNA dysregulation. Ducarre et al. (2024) showed modifications in FF-EV shape, size heterogeneity, and protein composition in infertile women with PCOS and DOR, indicating changes in EV formation pathways regulated by ESCRT machinery and Rab GTPases [79]. These physical anomalies may also influence EVs’ target selection and absorption efficacy by recipient cells. Gu et al. (2024) demonstrate that age-associated FF-EVs have reduced levels of TCA cycle intermediates and steroid precursors, correlating with the downregulation of ex-miRs that regulate enzymes such as IDH3, CYP11A1, and STAR. These metabolomic alterations, essential for oocyte competence and steroidogenesis, impair mitochondrial function and cholesterol transport [65].

Clinically, these ex-miR profiles can serve as non-invasive biomarkers for treatment personalization and patient stratification. Proposing their role as indicators of ovarian health, miR-21 and miR-34c in follicular fluid extracellular vesicles correlate positively with oocyte maturity, fertilization rates, and blastocyst development [88]. Conversely, elevated levels of miR-15a-5p, miR-143-3p, and miR-155-5p indicate a bad prognosis and may influence treatment strategies. The therapeutic alteration of the follicular ex-miR environment using synthetic miRNA mimics, antagomirs, or modified extracellular vesicles capable of reestablishing molecular balance and enhancing COS responsiveness may represent future avenues. These techniques offer a promising adjunct to existing ART therapies, particularly for those categorized as poor responders or those with PCOS experiencing refractory anovulation.

### 3.5. Ex-MiRs as Therapeutic Targets and Non-Invasive Biomarkers in ARTs

The integration of ex-miRs into clinical ART treatments signifies a notable progression in the quest for precision reproductive medicine. These diminutive regulatory RNAs are durable, readily observable, and physiologically significant, enclosed within the protective lipid bilayer of extracellular vesicles. Ex-miRs not only indicate pathophysiological changes but also function as active participants in illness progression or recovery, as they directly modify gene expression via post-transcriptional silencing processes, unlike conventional protein biomarkers [89]. They are released into the follicular fluid by granulosa cells, cumulus cells, and possibly the oocyte, allowing them to serve as real-time cellular activity reporters [90]. Ex-miRs serve as ideal candidates for real-time, non-invasive evaluation of follicular competency during ART cycles, as they reflect the varying hormonal, metabolic, and stress factors inside the follicular microenvironment.

Ex-miRs have been shown in many studies to act as predictive markers for oocyte quality, embryo viability, and pregnancy outcomes. In FF-EVs from follicles producing MII oocytes that eventually develop into high-quality embryos after fertilization, miR-21 and miR-34c are significantly enriched. By decreasing PTEN, miR-21 enhances survival, proliferation, and extracellular matrix remodeling in gastric and colorectal cancers, hence amplifying the PI3K–AKT pathway. This action is essential for meiotic competence and cytoplasmic maturation, guaranteeing that the oocyte exists in a metabolically conducive and anti-apoptotic milieu. Conversely, via inhibiting BCL2 and SIRT1, miR-34c modulates chromatin structure and apoptotic thresholds, therefore preserving oocyte DNA integrity and preparedness for fertilization. Benedetti et al. (2024) found that miR-34c mimics improve blastocyst quality when applied during in vitro maturation (IVM). Liquid biopsy techniques offer a less invasive means to evaluate oocyte developmental potential by identifying miRNAs in follicular fluid or maybe in plasma, influencing decisions on the timing of oocyte retrieval and the selection of embryo transfer. In contrast, increased levels of some ex-miRs have been definitively linked to poorer ART results and reduced follicular dynamics. The often raised ex-miRs in women with POR and PCOS encompass miR-15a-5p, miR-143-3p, and miR-155-5p; their biological effects include the induction of apoptosis, metabolic dysregulation, and diminished cumulus growth. MiR-15a-5p stimulates pro-apoptotic pathways by inhibiting BCL2, so initiating the mitochondrial death cascade and leading to GC depletion [47].

In ARTs, the therapeutic modulation of ex-miR activity represents an innovative frontier. Supplementing cultures with ex-miR mimics or antagomirs has shown in experimental trials the restoration of essential signaling pathways and enhancement of oocyte outcomes. Inhibition of miR-339-5p, which diminishes ERK1/2 signaling by SFPQ targeting, was demonstrated to promote CC development, nuclear maturation, and eventual BL formation (Han et al., 2024). The findings suggest that defective follicles can be reprogrammed using miRNA-based therapies, increasing their sensitivity to hormonal cues and so augmenting the probability of fertilization and implantation [50].

The progression of EV engineering technologies significantly improves the viability of ex-miR-based therapies. Extracellular vesicles can be bioengineered to sequester particular miRNAs, antisense oligonucleotides, or gene-editing tools like CRISPR-Cas9 to accurately modulate follicular signaling networks [91]. Mesenchymal stem cell-derived extracellular vesicles, loaded with anti-apoptotic or pro-metabolic microRNAs, co-cultured with cumulus–oocyte complexes, enhance oocyte competence. Furthermore, directing extracellular vesicles to particular ovarian compartments by surface ligands or aptamers may improve the accuracy and effectiveness of these therapies. Endogenous EVs provide an ideal basis for clinical translation owing to their diminished immunogenicity and enhanced biocompatibility relative to synthetic vectors. Their utilization in traditional IVF laboratories for supplementary therapy is further supported by their scalable production and storage capacities [92].

Diagnostic approaches for miRNA detection have progressed markedly. High-throughput methodologies like as short RNA sequencing, RT-qPCR arrays, and digital PCR may identify low-abundance ex-miRs with exceptional sensitivity and specificity [93]. Size-exclusion chromatography, ultracentrifugation, and microfluidic chips are enhancing the standardization of extracellular vesicle extraction from follicular fluid, serum, or urine through reproducible techniques. Ex-miR panels, including miR-21, miR-34c, miR-15a-5p, and miR-143-3p, have been suggested by several studies as diagnostic tools for categorizing patients according to expected ovarian response, embryo quality, and likelihood of conception.

The integration of these panels into ART cycles for real-time evaluation will provide adaptive adjustments in oocyte retrieval timing, stimulation dosage, and embryo transfer decisions.

## 4. Clinical Translation and Future Perspectives

Ex-miRs will be employed in ARTs to connect mechanistic discovery with therapeutic advancement. Notwithstanding the considerable increase in ex-miR content in FF-derived EVs, their regular clinical utilization requires additional standardization, mechanistic validation, and translational refinement. Ex-miRs both mirror and actively shape the cellular milieu of GCs, CCs, and the oocyte, so presenting a distinctive convergence of diagnostic relevance and functional significance. Their ability to modulate essential pathways like as PI3K–AKT, ERK1/2, mTOR, and TGF-β makes them important not just for assessing follicular state but also as direct targets for therapeutic intervention. The main goals of future initiatives should be the incorporation of technology methods, the identification of reliable ex-miR indicators, and the creation of efficient delivery systems for therapeutic modulation in real-time clinical environments.

Integrating EV isolation with ex-miR detection techniques is essential. Inter-laboratory variability is presently ascribed to FF processing, EV extraction methodologies (such as differential centrifugation and SEC), and ensuing quantification procedures (including RT-qPCR, sRNA-seq, and ddPCR). This challenges the validation of ex-miR signatures among patient populations. Standardizing methodology that employs synthetic spike-in controls, UMI barcoding, and stringent normalizing techniques would improve reproducibility and enable meta-analytical integration of ex-miR data. Moreover, collaborative multicenter studies that include clinical objectives such as MII oocyte rate, blastocyst development, and clinical pregnancy rate are crucial for defining actionable thresholds for ex-miRs, including miR-21, miR-143-3p, and miR-155-5p. Upon validation, these miRNAs may behave as universal indicators of follicular competency or dysfunction.

The therapeutic modulation of exosomal microRNAs, with diagnostics, signifies revolutionary progress. Administering designed extracellular vesicles carrying miRNA mimics (e.g., miR-424-5p, miR-34c) or inhibitors (e.g., anti-miR-15a-5p, anti-miR-339-5p) can accurately regulate aberrant signaling in polycystic ovarian reserve or polycystic ovary syndrome follicles. These medicines restore CDCA4 suppression, normalize ERK1/2 and PI3K–AKT pathways, and enhance GC viability, CC proliferation, and oocyte cytoplasmic maturation based on in vitro studies. Direct injection or IVM can be utilized to transfer EVs into growing follicles, facilitating selective reprogramming of the follicular niche. Combining EV therapy with metabolic adjuvants (e.g., myo-inositol, resveratrol) or hormonal drugs (e.g., FSH, LH) may improve oocyte competence and developmental potential. The application of this approach in clinical environments largely depends on progress in GMP-scale production, aptamer targeting, and lipid nanoparticle-mediated loading of extracellular vesicles.

Concurrently, systematic ex-miR profiling presents a non-invasive technique with considerable potential. Although FF-EVs offer a direct insight into the follicular milieu, circulating ex-miRs in serum or plasma may reflect more extensive ovarian and endocrine states. Investigations into serum miR-181a, miR-1248, and miR-27a have linked ovarian age, insulin resistance, and hormonal resistance—traits typically seen in decreased ovarian reserve and polycystic ovary syndrome—with alterations in these biomarkers. In spontaneous or constrained COS cycles where FF is not easily obtainable, serum-based tests may be advantageous for monitoring follicular changes. For patients with recurrent implantation failure or idiopathic infertility, early evaluation of oocyte quality and endometrial receptivity may prove advantageous.

A primary emphasis is on guiding embryo selection and synchronizing the endometrium by the application of ex-miRs. Modern judgments about embryo transfer predominantly rely on morphokinetics and, occasionally, preimplantation genetic testing for aneuploidy (PGT-A). These metrics, however, neglect functional or epigenetic oocyte quality. Ex-miRs in SCM linked to specific embryos may provide a non-invasive perspective on their metabolic, apoptotic, and developmental capabilities. Increased concentrations of miR-34c or miR-132 in SCM would signify epigenetic proficiency and correlate with BL quality. Exosomal microRNAs in uterine fluid, including miR-145 and miR-30d, may serve as predictors of endometrial receptivity and implantation readiness. Optimal implantation time and precise embryo matching may be attained by integrating ex-miR data from both the embryo and endometrial compartments.

It is imperative that ethical and legal frameworks develop in tandem with the research. Ex-miR-based diagnostics and therapies, especially concerning gametes or embryos, raise significant issues about safety, consent, and long-term implications in clinical practice. Regulatory agencies, including the EMA and FDA, will require criteria for the approval of ex-miR-based therapeutics that address pharmacokinetics, biodistribution, immunogenicity, and reproductive safety. Ethical guidelines must determine if modifications to ex-miRs during follicular or embryonic development qualify as germline modification and the optimal methods for educating patients about these approaches. Successful implementation must fundamentally rely on clear communication, patient-centered consent processes, and post-market surveillance.

Ultimately, advancement in this sector will require interdisciplinary cooperation. Reproductive biologists, doctors, molecular engineers, and computer scientists will collaboratively devise scalable techniques for the isolation, profiling, and analysis of ex-miRs. By employing ex-miR signatures, patient demographics, and treatment methodologies, AI and ML algorithms can be trained on large datasets to forecast ART outcomes. A thorough examination of ex-miR involvement in disease-specific situations, including endometriosis, obesity, or POI, will be enhanced by the establishment of biobanks holding annotated FF, serum, and SCM samples from varied populations. These methodologies will ultimately deliver tailored, molecularly guided reproductive medicines to replace empirical ARTs.

## 5. Discussion

Ex-miRs have emerged as pivotal regulators of intercellular communication within the follicular milieu, markedly affecting oocyte maturation, granulosa cell and cumulus cell functionality, as well as basement membrane formation [94]. Small ncRNAs within EVs serve as both biomarkers and active molecular agents in essential signaling networks. The synthesis of current literature underscores the importance of ex-miRs in the regulation of PI3K–AKT, ERK1/2, mTOR, TGF-β/SMAD, apoptotic, glycolytic, and cell cycle pathways. Their diagnostic and therapeutic efficacy in ARTs is supported by mechanistic research and clinical findings.

Multiple studies have discovered particular ex-miRs in follicular fluid that correlate with oocyte developmental competence. Jenabi et al. (2023) established that the effective suppression of PTEN, which promotes the continuous activation of the PI3K–AKT signaling pathway, resulted in a positive correlation between miR-21 expression in follicular fluid (FF) and cumulus cells (CCs) with the presence of MII oocytes and improved follicle recovery (FR). This stimulation causes phosphorylation of AKT at Ser473 and Thr308, thereby activating pathways that enhance cell cycle proliferation and survival while blocking pro-apoptotic proteins [29]. Benedetti et al. (2024) concurrently noted that miR-34c was markedly increased in FF-EVs sourced from follicles yielding competent oocytes. The use of mimic supplementation during IVM has been functionally validated, showing that miR-34c improves blastocyst quality by modulating BCL2, SIRT1, and cell cycle genes associated with chromatin remodeling and epigenetic stability. The findings suggest that miR-21 and miR-34c act as predictive indicators of oocyte competence and contribute functionally to the molecular regulation of folliculogenesis and the oocyte–embryo transition [47].

An anomalous ex-miR profile has been identified in PCOS, signifying significant changes in GC metabolism and follicular integrity. As previously stated, miR-143-3p and miR-155-5p are associated with the impairment of glycolytic metabolism and the decline of oocyte competence in polycystic ovarian syndrome (PCOS) [48]. Ex-miRs have been specifically associated with diminished follicular survival and oocyte maturation in DOR or POR. In POR patients, elevated levels of miR-15a-5p may promote apoptosis through the mitochondrial pathway by targeting BCL2 [40]. These molecular findings emphasize the significance of ex-miRs in regulating cell survival, senescence, and follicular competence, as well as their potential application in identifying and managing age-related or pathological ovarian dysfunction [49].

Ex-miRs modulate intracellular signaling in old follicles, clarifying the physiological deterioration in oocyte quality and developmental potential. Gu et al. (2024) discovered notable changes in exosomal metabolites linked to aging through the metabolomic analysis of FF-EVs in women of varying reproductive ages. The modified metabolites were intricately linked to hormonal pathways, including estradiol synthesis, as well as to nutrition sensing and redox signaling, both of which are essential for preserving oocyte competency. The age-related metabolic changes in FF-EVs suggest a reduction in the biochemical support necessary for proper oocyte maturation [65]. A considerable proportion of the 37 miRNAs discovered by Santonocito et al. (2014) as markedly enriched in FF-EVs targeted genes that suppress follicular activation and meiotic development, including FOXO1 and SMAD3. These targets are crucial in the processes that postpone oocyte maturation under unfavorable conditions, suggesting that the dysregulation of these miRNAs may impede timely follicular growth in older women. The interaction of metabolomic and transcriptomic dysregulation in FF-EVs underscores the complex role of ex-miRs in promoting the age-related deterioration of female fertility. The growing body of evidence underscores the significance of EV cargo in influencing oocyte quality via direct gene control and extensive metabolic programming within the ovarian follicle [11].

Mechanistic investigations employing animal models further validate the functional importance of ex-miRs in modulating oocyte maturation. In in vitro studies employing pig models, Han et al. (2024) found that FF-EVs substantially modified oocyte developmental dynamics. In these FF-EVs, miR-339-5p was identified as a direct target of SFPQ, a splicing factor involved in RNA processing and the control of ERK1/2 signaling. The strong expression of miR-339-5p reduced the phosphorylation of ERK1/2, a key regulator of cell cycle proliferation and meiotic resumption. The overexpression of MiR-339-5p resulted in compromised cumulus expansion, modified cortical granule distribution, decreased expression of genes linked to oocyte maturation, and eventually reduced rates of oocyte maturation and blastocyst formation. The diminution of miR-339-5p reinstated these molecular alterations, signifying a causal function. The research underscores the possibility of therapeutically modifying EVs’ cargo in IVM procedures to improve oocyte competency and illustrates how the dysregulation of a single ex-miR can trigger cascading effects across many maturation phases [50].

The data collectively underscore the crucial function of ex-miRs as active molecular regulators within the follicular niche. Ex-miRs regulate many intracellular signaling pathways inside their encapsulation in EVs, influencing GC and CC activity, metabolic cooperation, and meiotic competence [95]. Ex-miRs demonstrate direct mechanistic connections between signaling networks, including PI3K–AKT, ERK1/2, and Rb–E2F1, and the normal or pathological outcomes of the follicle, unlike traditional biomarkers. Their consistent presence in follicular fluid, serum, and synovial fluid makes them particularly appealing candidates for non-invasive ART diagnostics.

Moreover, functional evaluations in both human and animal models corroborate their therapeutic efficacy. In instances of PCOS and POR, engineered extracellular vesicles carrying miRNA mimics or inhibitors can be employed to address faulty pathways both in vitro and in vivo, thus establishing a basis for tailored ART therapies. To improve oocyte quality, restoring the impaired function of miR-339-5p or miR-155-5p may restart ERK1/2 signaling or glycolysis, respectively [96].

Nevertheless, specific obstacles remain despite these developments. Methodological discrepancies in extracellular vesicle isolation, insufficient consistency in microRNA quantification, and the limited size and diversity of study populations impede reproducibility and therapeutic translation. Furthermore, direct functional validation is still inadequately developed, despite the strong correlation between ex-miRs and reproductive outcomes. Future research must rectify these deficiencies through integrated omics analysis, comprehensive multi-center clinical trials with well-specified outcomes, and longitudinal observation of embryo development.

Ex-miRs represent a swiftly advancing field with considerable ramifications for reproductive biology. They enable the development of accurate ART regimens by linking molecular signals to clinical characteristics. The incorporation of ex-miR profiling into ART approaches enhances oocyte selection, embryo viability prediction, and eventually leads to enhanced reproductive outcomes, hence offering significant promise for customized infertility diagnosis and treatment.

## 6. Conclusions

This study highlights the significance of ex-miRs, conveyed through EVs in FF, as essential molecular regulators of follicular development, oocyte maturation, and early embryonic viability. Ex-miRs regulate the functional efficacy of CCs, GCs, and ultimately the oocyte by modulating essential intracellular pathways, including PI3K–AKT, ERK1/2, mTOR, TGF-β/SMAD, as well as apoptotic and glycolytic pathways. Data produced under physiological and pathological conditions, such as PCOS, DOR, and POR demonstrates how specific ex-miRs can enhance or impair cellular function, metabolic cooperation, and meiotic progression.

Ex-miRs serve dual purposes: they may act as therapeutic targets to enhance ARTs outcomes and function as diagnostic indicators for assessing oocyte and embryo viability. Their stable, extracellular nature facilitates little invasive detection in FF, serum, and SCM, hence permitting integration into clinical procedures. Furthermore, preclinical studies suggest that altering ex-miR expression or designing EVs cargo may enhance oocyte production in poor ovarian response (POR), correct abnormal signaling in PCOS, and facilitate in vitro maturation (IVM).

Prioritizing the standardization of EVs isolation and miRNA quantification methodologies is essential for future research; validation studies must encompass a broader range of patient populations; and the therapeutic effectiveness of ex-miR-targeted interventions should be evaluated. Precision reproductive medicine is fundamentally enabled by the incorporation of ex-miR profiling into assisted reproductive technologies, significantly improving diagnostic accuracy and therapeutic personalization in infertility treatment.

## Figures and Tables

**Table 1:** Functional Roles and Clinical Relevance of Key Exosomal miRNAs in Human Follicular Fluid.

Exosomal miRNA	Primary Target(s)	Pathway Modulated	Effect on Follicular Function	Clinical Relevance
miR-21, [29]	PTEN	PI3K–AKT	Promotes CC survival and oocyte maturation	Predictor of oocyte competence
miR-34c, [47]	BCL2, SIRT1	Apoptosis, Epigenetic regulation	Improves BL quality and oocyte epigenetic stability	BL development biomarker
miR-15a-5p, [40]	BCL2	Mitochondrial apoptosis	Induces GC apoptosis in POR	Marker of POR
miR-143-3p, [48]	HK2, PFKM	Glycolysis	Suppresses metabolic support in PCOS	Metabolic dysfunction in PCOS
miR-155-5p, [48]	FADD, Caspase-3, BIM	Apoptosis	Reduces GC viability in PCOS	GC health indicator in PCOS
miR-424-5p, [49]	CDCA4	Rb–E2F1 Cell Cycle	Reverses GC senescence	Therapeutic target in PCOS
miR-339-5p, [50]	SFPQ	ERK1/2	Impairs CC expansion and oocyte quality	Candidate for IVM intervention

In the framework of ARTs, this table describes certain ex-miRs found in FF together with their known molecular targets, signaling pathways, effects on granulosa and cumulative cell function, and clinical relevance. Key regulatory networks controlling oocyte maturation, metabolic support, apoptosis resistance, and follicular senescence link miRNAs mentioned below; they might also be therapeutic targets or biomarkers.
